# The characteristics of serum lipid spectrum in PanNENs and its correlation with clinicopathological features and prognosis

**DOI:** 10.3389/fendo.2023.1137911

**Published:** 2023-03-24

**Authors:** Yu-Fan Meng, Zhi-Yao Fan, Jian Yang, Yong-Zheng Li, Shu-Jie Liu, Chang-Hao Gao, Xin Gao, Chao-Yu Pang, Han-Xiang Zhan

**Affiliations:** Division of Pancreatic Surgery, Department of General Surgery, Qilu hospital, Shandong University, Jinan, Shandong, China

**Keywords:** hyperlipidemia, insulinoma, lipids, pancreatic neuroendocrine neoplasms, pancreatic tumor

## Abstract

**Background:**

The role of dyslipidemia in pancreatic neuroendocrine tumors (PanNENs) is unclear. The aim of this study is to analyze the characteristics of serum lipid spectrum in PanNENs, and the effect of the variation in lipid profile on the development of PanNENs clinicopathological features and prognosis.

**Methods:**

All PanNENs patients between November 2012 and September 2020 in the authors’ research center were identified from patient medical records and databases. A total of 185 with PanNENs patients were ultimately included in this study, including 100 nonfunctional PanNENs and 85 insulinomas. Clinicopathologic features, serum lipid level and overall survival results were retrospectively analyzed using statistical methods.

**Results:**

In 185 PanNENs, 95 (51.4%) patients appear to have dyslipidemia. Patients with insulinoma had a lower proportion of abnormal HDL than those with nonfunctional PanNENs (10.6% vs 23%, P=0.026). The mean serum HDL levels of insulinomas were 0.131 mmol/L higher than the NF-PanNENs (1.306 ± 0.324 vs 1.175 ± 0.315, P=0.006). In multivariate logistic analysis, high levels of HDL are negatively correlated to tumor size (OR 0.233, 95% CI: 0.069-0.790, P=0.019), but HDL was not associated with pathological grade or metastasis. And a correlation has been found between hypercholesterolemia and the original location of the tumor (OR:0.224, 95%CI: 0.066-0.753, P =0.016). In addition, the outcome of the survival analysis revealed that dyslipidemia did not influence the prognosis of PanNENs patients (P>0.05).

**Conclusions:**

HDL was negatively correlated with the tumor size of PanNENs. The serum HDL level of insulinoma patients is higher than nonfunctional PanNENs.

## 1 Introduction

Pancreatic neuroendocrine neoplasms (PanNENs) are a group of highly heterogeneous solid tumors derived from endocrine tissues of the pancreas (1). Although PanNENs are relatively rare and account for only less than 5% of all pancreatic tumors, the rate of PanNENs has significantly risen over the past 20 years (2–4). In general, PanNENs are classified into functional and nonfunctional owing to their secretion of hormones (5, 6), which show differences dramatically in histology and biological behaviors. PanNENs have different performances on development, prognosis, and clinical manifestations. The early identification and therapeutic intervention contribute to preventing tumor metastasis and improving the prognosis of PanNENs (7). Thus, it is essential to identify potential novel biomarkers to improve the diagnosis and prognostic of PanNENs.

A large number of clinical studies have demonstrated that abnormal lipid levels and lipoprotein are related to various malignant tumors’ occurrence, progression, and prognosis (8–11). A 2018 meta-analysis published by Zhou and colleagues included 24,655 participants who argued that total cholesterol (TC) and high-density lipoprotein cholesterol (HDL) were protective factors (12), and high-level HDL and TC markedly reduces the risk of recurrence and mortality in cancer patients. HDL may modulate cytokine production and reduce oxidative stress to exert protective effects (13, 14). TC is known to play a crucially important role in cellular structure and function. Preliminary studies have indicated that excess dietary cholesterol intake prominently rich the risk of pancreatic cancer but is not related to its prognosis (11, 15). Nevertheless, the anti-cancer effects of serum lipids and lipoproteins remain controversial and the mechanism is unclear. In functional PanNENs, insulinomas are the most common type. Insulinoma cells can sustain the release of insulin and are not affected by blood glucose levels, which often leads to frequent episodes of hypoglycemia (16, 17). Abnormal glucose metabolism is often concomitant with dyslipidemia such as diabetes and generally causes elevated levels of plasma triglycerides (18, 19). At present, whether and how there is a difference in blood lipid levels between insulinomas and nonfunctional PanNENs (NF- PanNENs) remains to be not investigated. Moreover, the influence of hyperlipidemia on the incidence and clinicopathological features of PanNENs remains poorly understood.

Consequently, this study retrospectively analyzed the blood lipid metabolism spectrum of PanNENs patients and statistically assessed the clinicopathological features and prognosis of patients, to explore the difference between insulinomas and NF-PanNENs in lipid level and the relationship between dyslipidemia and clinicopathological features of PanNENs.

## 2 Methods and materials

### 2.1 Patient selection

**Figure 1:**
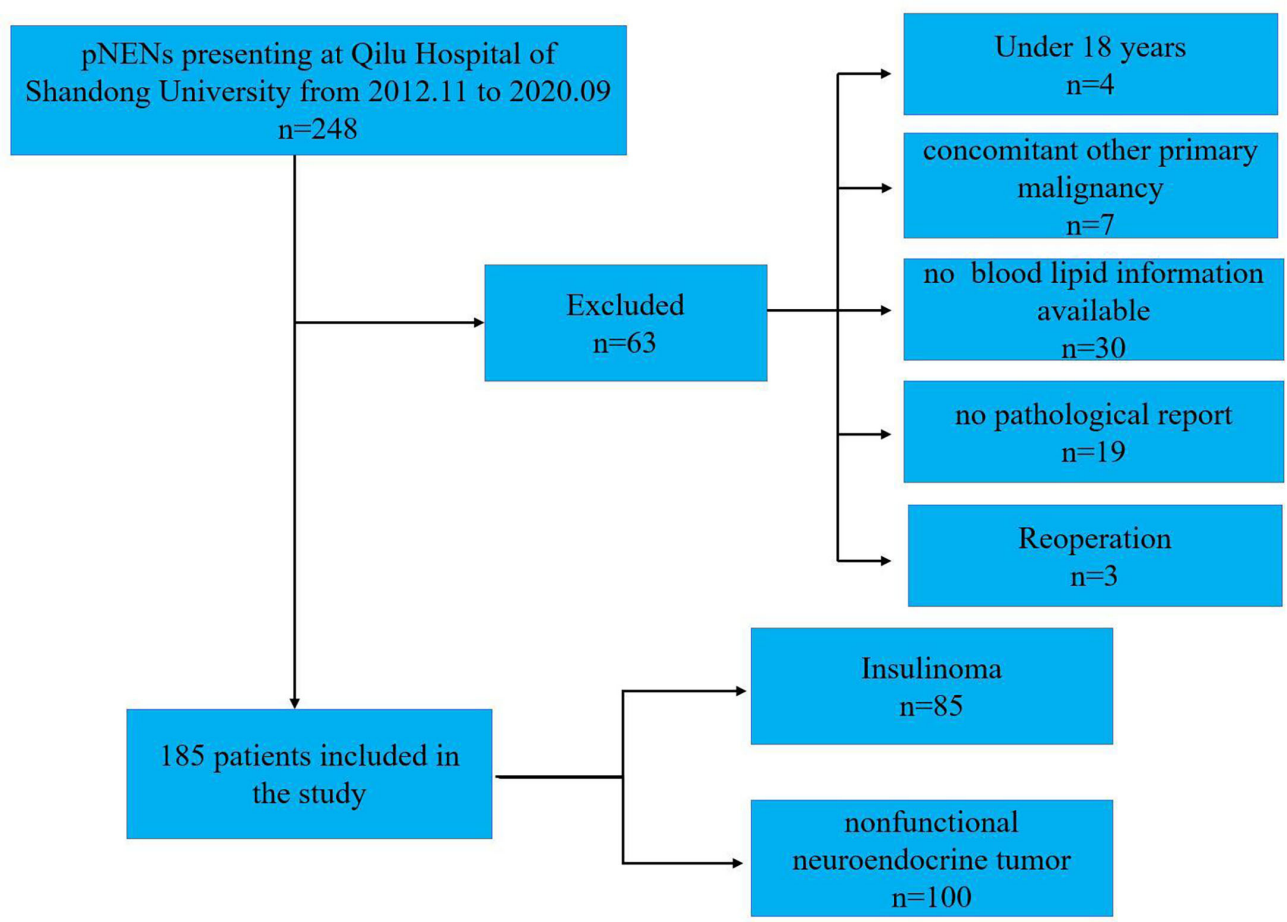
Descriptive flow chart for inclusion and exclusion of patients in this study.

This research retrospectively analyzed the records of the 248 PanNENs patients from November 2012 to September 2020 at Qilu Hospital of Shandong University, and all patients were confirmed as PanNENs according to histologic or cytologic evidence. The study protocol has been approved by the ethics committee of Qilu Hospital of Shandong University, and the total of the PanNENs patients agreed to their data being used for research. The exclusion criteria were as follows (1): without the report of TC, TG (triglyceride), HDL, and LDL (low-density lipoprotein) before treatments (2); concomitant other malignant tumors diagnosed; (3) patients with endocrine diseases or other than dyslipidemia, e g. hypercortisolism; (4) absence or hypoplasia of clinicopathological data; and (5) patients under the age of 18. Ultimately, 63 patients not meeting these criteria were removed, and a total of 185 with PanNENs were included in this study (**Figure 1**).

### 2.2 Data collection

All patients met the following eligibility criteria: (1) all patients had pathologically confirmed neuroendocrine tumors; (2) without cancer history;(3) no treatment before admission. Collecting serum collection before receiving treatment, the profiles of serum lipids, including TC, HDL, LDL, and TG, were measured using Cobas 8000(701) after at least 12 hours of fasting.The blood samples of all patients were uniformly processed by the clinical laboratory of Qilu Hospital and the test results were obtained. Baseline clinical data, including age, sex, body mass index (BMI), tumor location, tumor size, presence or absence of distant metastasis, tumor grade, and blood lipid data were assembled from the patients’ medical records from data of Qilu Hospital of Shandong University. The tumor grade was determined according to the 2019 WHO classification of tumors of the digestive system (20). NETs G3 and NECs were divided in terms of their immunohistochemistry, including Ki-67, P53, Rb and other indicators (21). According to whether causes a clinical syndrome, PanNENs is divided into NF-PanNENs and functional PanNENs (22). In this study, we focused on insulinoma among functional neuroendocrine tumors. TNM staging was performed based on the eighth version of the American Joint Committee on Cancer Staging Manual (6). The prognosis information of patients was obtained through outpatient service and telephone follow-up, last accessed April 01, 2022. Overall survival (OS) was defined as the time from the time of the definite diagnosis of PanNENs to the time of death.

Diagnostic criteria of dyslipidemia were determined based on the guidelines for the treatment and prevention of dyslipidemia revision (23). In this study, dyslipidemia was diagnosed by meeting at least 1 of the 4 following criteria: TC ≥ 5.2mmol/L, triglyceride, TG ≥ 1.70mmol/L, HDL < 1mmol/L, and LDL ≥ 3.4mmol/L.

### 2.3 Statistical analysis

All statistical analyses were conducted using IBM SPSS Statistics version 25. The independent sample t-tests were conducted for normally distributed data and Mann-Whitney U tests were performed for nonnormally distributed data. Fisher and Pearson χ2 tests were applied to categorical parameters. Logistic regression analysis was used to analyze the correlations between serum lipid and clinicopathological features, such as sex, age, tumor location, tumor diameter, and distant metastasis. The strength of the association between clinicopathological features and dyslipidemia was estimated with odds ratios (OR). The Kaplan-Meier and log-rank tests were used to calculate survival curves and to compare differences. In the survival analysis, the 27 PanNENs patients who were lost to follow-up were excluded. Using cox proportional hazards models, multivariate analyses and test independent significance variables were conducted. Hazard ratio (HR) was reported as relative risk and statistical stability was evaluated with 95% confidence intervals. A p-value<0.05 was considered statistically significant.

## 3 Results

### 3.1 Clinicopathologic characteristics of insulinomas and NF-PanNENs

A total of 185 with PanNENs patients were ultimately included in this study, including 100 NF-PanNENs and 85 insulinomas (**Figure 1**). The median age was 53 (range:18–82 years), and insulinoma shows a trend of female preponderance compared to NF-PanNENs (65.9% vs 52%, P=0.056) (**Table 1**). A total of 56 lesions were observed in the insulinoma body and tail. 82.4% (70/85) of insulinomas were less than 2cm in diameter. Of total of 100 NF-PanNENs, 16 patients presented with distant metastasis at diagnosis. As for the clinical staging and pathological grade, the insulinoma patients were mainly clinical phase I and II and were G1 and G2 stage. And seventeen percent of NF-PanNENs patients were diagnosed with G3 and neuroendocrine carcinoma (NEC), which was fourteen times greater than insulinoma patients (P<0.001). Notably, although slightly more than half of insulinoma patients are overweight (BMI≥ 25 kg/m^2^), there was no significant difference between NF-PanNENs and insulinomas in the proportion of hyperlipidemia, and none of the TC, TG, and LDL differences were statistically significant (P> 0.05). However, the proportion of NF-PanNENs patients with an abnormal HDL (<1mmol/L) number was more than twice as high as in insulinoma patients (23.0% vs 10.6%, P=0.026) (**Table 1**).

**Table 1:** Clinicopathological characteristics of patients with insulinoma and nonfunctional PanNENs.

Parameters	N	Nonfunctional PanNENs	Insulinoma	P-value
Total	185	100	85	
**Age, n (%)**				0.704
<60 years	128	68 (68)	60 (70.6)	
≥60 years	57	32 (32)	25 (29.4)	
**Gender, n (%)**				0.056
Female	108	52 (52)	56 (65.9)	
Male	77	48 (48)	29 (34.1)	
**BMI ^a^, n (%)**				0.001
< 25 kg/m^2^	74	49 (72.1)	25 (43.1)	
≥ 25 kg/m^2^	52	19 (27.9)	33 (56.9)	
**Tumor location ^b^, n (%)**				0.002
Head and Neck	78	52 (55.3)	26 (31.7)	
Body and Tail	98	42 (44.7)	56 (68.3)	
**Tumor size ^c^, n (%)**				< 0.001
≤ 2cm	93	23 (24.2)	70 (82.4)	
> 2cm	87	72 (75.8)	15 (17.6)	
**Pathological grade^d^, n (%)**				< 0.001
G1 and G2	163	83 (83)	80 (98.8)	
G3 and NEC	18	17 (17)	1 (1.2)	
**Distant metastasis, n (%)**				0.006
Absence	166	84 (84)	82 (96.5)	
Presence	19	16 (16)	3 (3.5)	
**Clinical stage, n (%)**				< 0.001
I and II	156	74 (74)	82 (96.5)	
III and IV	29	26 (26)	3 (3.5)	
**Hyperlipidemia, n (%)**				0.627
Absence	90	47 (47)	43 (50.6)	
Presence	95	53 (53)	42 (49.4)	
**TC, n (%)**				0.537
<5.2 mmol/L	141	78 (78)	63 (74.1)	
≥5.2 mmol/L	44	22 (22)	22 (25.9)	
**TG, n (%)**				0.723
<1.7 mmol/L	137	73 (73)	64 (75.3)	
≥1.7 mmol/L	48	27 (27)	21 (24.2)	
**HDL, n (%)**				0.026
<1.0 mmol/L	32	23 (23)	9 (10.6)	
≥1.0 mmol/L	153	77 (77)	76 (89.4)	
**LDL, n (%)**				0.688
<3.4 mmol/L	159	85 (85)	74 (87.1)	
≥3.4 mmol/L	26	15 (15)	11 (12.9)	

The categorical data was analyzed with Pearson Chi-square Test. ^a^ 27 patients with insulinoma and 32 patients with nonfunctional PanNENs lacked height and weight data, so their BMI could not be obtained. ^b^ 3 insulinoma patients and 6 patients with nonfunctional PanNENs were unable to determine the tumor location due to multiple tumors, ^d^ 4 insulinoma patients could not be classified by pathology due to the lack of Ki-67 index and mitotic index, ^c^ 5 patients with nonfunctional PanNENs lacked the maximum diameter of the tumor. TG, triglyceride; TC, total cholesterol; HDL, high-density lipoprotein; LDL, low-density lipoprotein.

### 3.2 Clinicopathologic characteristics of PanNENs with different serum lipid profile

Ninety-five PanNENs patients appear dyslipidemia, comprising seventeen patients were hypertriglyceridemia, four patients were hypercholesterolemia, and nineteen patients were low HDL, and two patients were high LDL. Fifty-three patients were complicated with two or more kinds of dyslipidemia. The statistical analysis indicates that there was no statistical difference between hyperlipidemia patients and the normal population in gender, age, tumor location, tumor size, distant metastasis, and pathological grade (P > 0.05) (**Table 2**). The detailed results between the level of HDL and clinical features were shown in **Table 3**. Only nine insulinoma patients appear abnormal HDL, and the overall rate of HDL values greater than 1 mmol/L was higher in insulinoma than in NF-PanNENs(89.4% vs 77%, P = 0.026). Tumors over 2cm tended to be a higher proportion when the serum HDL level was under 1 mmol/L (65.5% vs 45%, P=0.043). Additionally, a smaller number of women were found to have abnormal HDL (P=0.002). However, no statistically significant difference in tumor location, pathological grade and clinical classification was found between low HDL patients and healthy people (P>0.05) (**Table 3**). Hypercholesterolemia and normal total cholesterol patients (P=0.042) have a significant difference in age. In all 44 patients with hypercholesterolemia, 72.7% were female and 27.3% male (P=0.027). The percentage of TC≥5.2 mmol/L increased from 27% in people under 60 years old to 43.2% in people over 60 years old (P=0.042), but no discrepancy with other factors (**Supplementary Table 1**).

**Table 2:** Comparison of clinicopathological parameters in PanNENs patients with normal or hyperlipidemia.

Parameters	N	Normal	Hyperlipidemia	p-value
Total	185	90	95	
**Age, n (%)**				0.384
<60 years	128	65(72.2)	63(66.3)	
≥60 years	57	25(27.8)	32(33.7)	
**Gender, n (%)**				0.663
Female	108	54(60)	54(56.8)	
Male	77	36(40)	41(43.2)	
**BMI ^a^, n (%)**				0.609
< 25kg/m^2^	74	39(60.9)	35(56.5)	
≥ 25kg/m^2^	52	25(39.1)	27(43.5)	
**Tumor location** ^b^ **, n (%)**				0.612
Head and Neck	78	36(42.4)	42(46.2)	
Body and Tail	98	49(57.6)	49(53.8)	
**Tumor size** ^c^ **, n (%)**				0.996
≤2cm	93	46(51.7)	47(51.6)	
> 2cm	87	43(48.3)	44(48.4)	
**Pathological grade^d^, n (%)**				0.384
G1 and G2	163	81(92)	82(88.2)	
G3 and NEC	18	7(8)	11(11.8)	
**Distant metastasis, n (%)**				0.069
Absence	166	77(85.6)	89(93.7)	
Presence	19	13(14.4)	6(6.3)	
**Clinical stage, n (%)**				0.115
I and II	156	72(80)	84(88.4)	
III and IV	29	18(20)	11(11.6)	
**Type, n (%)**				0.627
Nonfunctional PanNENs	100	53(53)	47(47)	
Insulinoma	85	42(49.4)	43(50.6)	

The comparison between normal blood lipid level and hyperlipidemia was performed using the Pearson Chi-square test method. Among the patients with hyperlipidemia,c 4 cases lacked the maximum diameter and b location of the tumor, ^a^ 33 cases could not calculate BMI due to lack of specific data such as height and weight, ^d^ 2 cases could not get accurate pathological grading due to incomplete pathological information. Among the patients with normal blood level, ^c^ 1 cases lacked tumor size, and ^b^ 5 cases could not be identified location due to multiple tumors, a 26 cases could not be calculated due to lack of specific data such as height and weight, d and 2 case could not be accurately classified due to incomplete pathological information.

**Table 3:** Comparison of clinicopathologic parameters of HDL.

Parameters	HDL <1mmol/L (n=33)	HDL ≥ 1mmol/L (n=152)	p-value
**Type, n (%)**			**0.026**
Nonfunctional PanNENs	23 (23)	77 (77)	
Insulinoma	9 (10.6)	76 (89.4)	
**Age, n (%)**			0.367
<60 years	20 (62.5)	108 (70.6)	
≥60 years	12 (37.5)	45 (29.4)	
**Gender, n (%)**			**0.002**
Female	11 (34.3)	97 (63.4)	
Male	21(65.6)	56(36.6)	
**BMI, n (%)**			0.380
< 25kg/m^2^	16 (66.7)	58 (56.9)	
≥ 25kg/m^2^	8 (33.3)	44 (43.1)	
**Tumor location, n (%)**			0.275
Head and Neck	16 (53.3)	62 (42.5)	
Body and Tail	14 (46.7)	84 (57.5)	
**Tumor size, n (%)**			**0.043**
≤2cm	10 (34.5)	83 (55)	
> 2cm	19 (65.5)	68 (45)	
**Pathological grade, n (%)**			0.530
G1 and G2	28 (87.5)	135 (90.6)	
G3 and NEC	4 (12.5)	14 (9.4)	
**Distant metastasis, n (%)**			1
Absence	29 (90.6)	137 (89.5)	
Presence	3 (9.4)	16 (10.5)	
**Clinical stage, n (%)**			0.289
I and II	25 (78.1)	131 (85.6)	
III and IV	7 (21.9)	22 (14.4)	

p-value from chi-square test. HDL, high-density lipoprotein. The bold indicates the difference was statistically significant (p<0.05).

### 3.3 Changes in PanNENs lipid levels

Based on the analysis of the t-test, the female group had remarkably higher HDL (1.322 ± 0.329 vs 1.114 ± 0.278, P<0.001) (**Table 4**). Results also display that the mean HDL level of tumors with diameter ≤2cm was lower than that of tumors with diameter > 2cm (1.300 ± 0.298 vs 1.189 ± 0.334, P=0.019) (**Table 4**). And the location of the tumor and the patient’s age don’t have any difference in the HDL profile. These results are the same as those for categorical variables. Combined with the average value, the serum mean HDL levels of insulinomas were higher 0.131 mmol/L than NF-PanNENs (P=0.006), and the differences of TC, TG and LDL were not statistically significant. To understand the difference in HDL levels in two types of PanNENs, the data were divided into NF-PanNENs and insulinoma for further analysis (**Table 5**). There are commonly higher HDL levels in the insulinoma than NF-PanNENs, specifically in over 60 ages, female, and normal BMI showed significant statistical differences (P < 0.05). In terms of tumor location, the mean HDL serum level of patients with insulinomas was 0.208 mmol/L higher than the mean HDL blood level of patients with NF-PanNENs between patients whose tumors are located in the head and neck (P <0.001). In **Table 4**, there is no statistically significant difference between low-grade (G1 and G2) and early-stage (I and II clinical classification) PanNENs on HDL levels, despite having elevated levels of HDL (P > 0.05). In **Table 5**, insulinomas and non-functional tumors only show variations in HDL levels in early-stage and low-grade tumors. In addition, TC (4.651 ± 1.057 vs 4.323 ± 1.125, P=0.044) levels than the male group, but there no exist obviously differences in serum TG and LDL on gender and age (**Supplementary Table 2**). TG mean levels show a clearly reduce when PanNENs were worse clinical stage (1.461 ± 0.850 vs 1.051 ± 0.383, P<0.001) (**Supplementary Table 2**). Similarly, there are no parameters that show a variation on the level of LDL (**Supplementary Tables 2. 3**).

**Table 4:** Comparison of clinicopathologic parameters of HDL level.

Parameters	HDL (mmol/L) `x ± s	p-value
**Type**		**0.006**
Nonfunctional PanNENs	1.175 ± 0.315	
Insulinoma	1.306 ± 0.324	
**Age**		0.799
<60 years	1.239 ± 0.307	
≥60 years	1.226 ± 0.364	
**Gender**		< **0.001**
Female	1.322 ± 0.329	
Male	1.114 ± 0.278	
**BMI**		0.234
< 25kg/m^2^	1.179 ± 0.305	
≥ 25kg/m^2^	1.243 ± 0.279	
**Tumor location**		0.527
Head and Neck	1.222 ± 0.369	
Body and Tail	1.254 ± 0.295	
**Tumor size**		**0.019**
≤2cm	1.300 ± 0.298	
>2cm	1.189 ± 0.334	
**Pathological grade**		0.447
G1 and G2	1.237 ± 0.319	
G3 and NEC	1.176 ± 0.368	
**Distant metastasis**		0.316
Absence	1.243 ± 0.326	
Presence	1.164 ± 0.317	
**Clinical stage**		0.144
I and II	1.250 ± 0.325	
III and IV	1.154 ± 0.314	

p-value from independent-sample t-test. HDL, high-density lipoprotein. The bold indicates the difference was statistically significant (p<0.05).

**Table 5:** Comparison of HDL levels in PanNENs with different clinicopathological parameters.

Parameters	HDL (mmol/L) 'x ± s	p-value
Age		
**<60 years**		0.067
Nonfunctional PanNENs	1.193 ± 0.287	
Insulinoma	1.292 ± 0.323	
**≥60 years**		**0.035**
Nonfunctional PanNENs	1.137 ± 0.368	
Insulinoma	1.340 ± 0.331	
Gender		
**Female**		**0.033**
Nonfunctional PanNENs	1.252 ± 0.318	
Insulinoma	1.387 ± 0.329	
**Male**		0.292
Nonfunctional PanNENs	1.085 ± 0.285	
Insulinoma	1.151 ± 0.249	
BMI		
**< 25kg/m^2^**		**0.016**
Nonfunctional PanNENs	1.119 ± 0.252	
Insulinoma	1.298 ± 0.366	
**≥ 25kg/m^2^**		0.314
Nonfunctional PanNENs	1.191 ± 0.298	
Insulinoma	1.273 ± 0.268	
Tumor location		
**Head and Neck**		< **0.001**
Nonfunctional PanNENs	1.130 ± 0.377	
Insulinoma	1.406 ± 0.368	
**Body and Tail**		0.709
Nonfunctional PanNENs	1.241 ± 0.288	
Insulinoma	1.264 ± 0.303	
Tumor size		
**≤2cm**		0.406
Nonfunctional PanNENs	1.255 ± 0.238	
Insulinoma	1.315 ± 0.315	
>**2cm**		0.594
Nonfunctional PanNENs	1.178 ± 0.323	
Insulinoma	1.224 ± 0.352	
Pathological grade		
**G1 and G2**		**0.022**
Nonfunctional PanNENs	1.181 ± 0.308	
Insulinoma	1.295 ± 0.322	
Distant metastasis		
**Absence**		**0.017**
Nonfunctional PanNENs	1.184 ± 0.317	
Insulinoma	1.304 ± 0.325	
**Presence**		0.231
Nonfunctional PanNENs	1.126 ± 0.306	
Insulinoma	1.370 ± 0.360	
Clinical stage		
**I and II**		**0.029**
Nonfunctional PanNENs	1.191 ± 0.318	
Insulinoma	1.304 ± 0.325	
**III and IV**		0.215
Nonfunctional PanNENs	1.129 ± 0.306	
Insulinoma	1.370 ± 0.360	

p-value from independent-sample t-test. HDL, high-density lipoprotein. The bold indicates the difference was statistically significant (p<0.05).

### 3.4 Correlation between serum lipid and clinicopathologic factors

According to the result of the multivariate logistic regression (**Table 6**), high-level HDL had a lower risk of developing tumor diameter over 2cm compared to patients with HDL levels below 1.0 mmol/L,and the result was statistically significant (OR:0.233, 95%CI:0.069-0.790, P=0.019). HDL level appears to correlate with tumor location (OR:3.609, 95%CI:0.981-13.277, P=0.053). In addition, in PanNENs patients, hypercholesterolemia can also be regarded as independent associated factors for tumors occurring in the pancreatic body and tail (OR:0.224, 95%CI: 0.066-0.753, P =0.016), but not for other clinicopathological parameters. Dyslipidemia, LDL, and TG levels were not independent risk factors for clinicopathological parameters, including tumor size, tumor loaction, metastasis**s**and pathological grading (P > 0.05) (**Table 6**).

**Table 6:** The associations between serum lipids changes and clinicopathologic parameters in PanNENs.

Parameters	Tumor location		Tumor size		Metastasis		Pathological grade	
	OR (95%CI)	p-value	OR (95%CI)	p-value	OR (95%CI)	p-value	OR (95%CI)	p-value
Hyperlipidemia								
NO	1		1		1		1	
YES	2.844(0.717-11.286)	0.137	0.416(0.116-1.494)	0.179	0.082(0.005-1.373)	0.082	1.277(0.181-9.025)	0.807
TC								
<5.2mmol/L	1		1		1		1	
≥5.2mmol/L	0.224(0.066-0.753)	**0.016**	1.284(0.438-3.763)	0.648	2.242(0.247-20.309)	0.473	2.139(0.415-11.022)	0.363
TG								
<1.70mmol/L	1		1		1		1	
≥1.70mmol/L	0.820(0.327-2.056)	0.672	1.335(0.524-3.406)	0.545	1.337(0.248-7.217)	0.736	0.850(0.223-3.235)	0.811
HDL								
<1mmol/L	1		1		1		1	
≥1mmol/L	3.609(0.981-13.277)	0.053	0.233(0.069-0.790)	**0.019**	0.167(0.012-2.314)	0.182	0.796(0.139-4.557)	0.797
LDL								
<3.4mmol/L	1		1		1		1	
3.4mmol/L	3.609(0.430-3.821)	0656	1.727(0.596-5.004)	0.314	2.708(0.224-32.679)	0.433	0.456(0.079-2.646)	0.382

OR, odds ratio; 95% CI, 95% confidence interval; TG, triglyceride; TC, total cholesterol; HDL, high-density lipoprotein; LDL, low-density lipoprotein. The bold indicates the difference was statistically significant (p<0.05).

### 3.5 Survival

**Figure 2:**
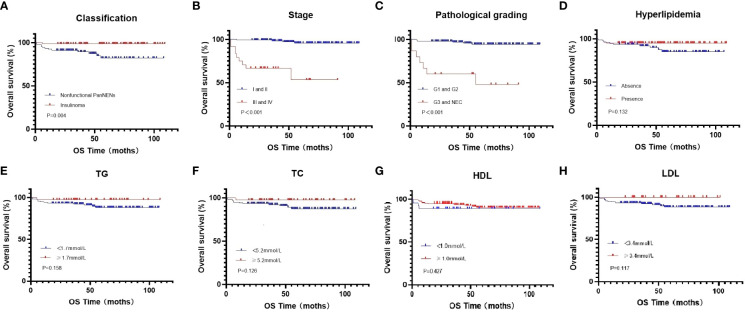
Kaplan-Meier curves for OS in all PanNENs patients **(A)**, regarding nonfunctional PanNENs vs insulinomas (P=0.004) **(B)**, regarding clinical phase clinical phase I and II vs clinical phase III and IV (P <0.001) **(C)**, regarding G1 and G2 vs G3 and NEC (P <0.001) **(D)**, regarding hyperlipidemia vs normal serum lipids (P =0.132) **(E)**, regarding low vs high TG levels (P =0.158) **(F)**, regarding low vs high TC levels (P =0.126) **(G)**, regarding low vs high HDL levels (P =0.427) **(H)**, regarding low vs high LDL levels (P =0.117).

**Figure 3:**
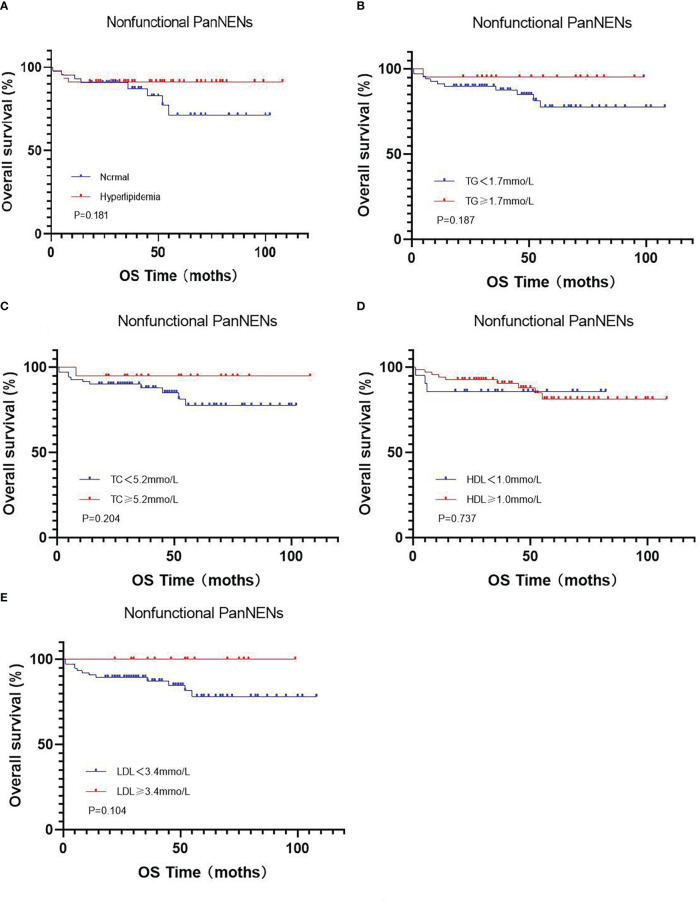
Kaplan-Meier curves for OS in the subgroup of nonfunctional PanNENs **(A)**, regarding hyperlipidemia vs normal serum lipids (P =0.181) **(B)**, regarding low vs high TG levels (P =0.187) **(C)**, regarding low vs high TC levels (P =0.204) **(D)**, regarding low vs high HDL levels (P =0.737) **(E)**, regarding low vs high LDL levels (P =0.104).

At the end of this follow-up period, 27 patients were lost to follow up and 13 of 158 (8.2%) patients with PanNENs had died. The median duration of all patients with PanNENs was 52 months (1–109 months), and the death rate in patients with normal and abnormal blood lipid levels were 8/80(10%) and 5/78 (6.4%), respectively. The median overall survival of follow-up in patients with normal and abnormal blood lipid levels was 52 months (1–107 months) and 51.5 months (1–109 months), respectively. Moreover, the effects of hyperlipidemia on the overall survival of PanNENs patients were examined in Kaplan–Meier plotter, and which results indicated no Statistical difference (P=0.132) (**Figure 2D**). We also separately analyzed the effect of TC, TG, LDL, and HDL on survival. Although the average OS of patients with PanNENs in TG(**Figure 2E**), TC (**Figure 2F**), HDL (**Figure 2G**), and LDL(**Figure 2H**) are different, their results are consistent with the results of dyslipidemia (P > 0.05). 1 of 69 (1.4%) patients with insulinomas and 12 of 89 (13.5%) patients with NF-PanNENs died. Besides, the early clinical stage (**Figure 2B**) and low pathological grade (**Figure 2C**) was remarkably associated with better prognosis. Patients with NF-PanNENs showed worse OS than insulinomas (mean OS: 63.0 vs 46.8 months, P =0.004) (**Figure 2A**). The effect of blood serum changes on NF-PanNENs were further analyzed. Although the Kaplan-Meier survival curves seem to have a separated trend, there is without statistical difference (**Figure 3**). By univariate analysis, insulinoma patients contributed to the 91% decline of mortality risk compared to NF-PanNENs, but not with multivariate analysis. In univariate and multivariate analyses for OS, age, pathological grading, and clinical staging can be used as independent predictors of OS in patients with PanNENs. This means patients under 60 years old (HR: 4.752, 95 % CI: 1.241-18.191, P = 0.023), well-differentiated PanNENs (HR: 5.180, 95 % CI:1. 309-20.493, P = 0.019) and clinical phase I and II staging (HR: 6.683, 95 % CI: 1.429-31.263, P = 0.016) have a better prognosis. However, serum lipid changes were not related to death risk (**Table 7**).

**Table 7:** Univariate and multivariate Cox proportional analysis with overall survival.

Characteristics	Univariate analysis			Multivariate analysis		
	HR	95%CI	p-value	HR	95%CI	p-value
Age						
<60 years	1					
≥60 years	5.975	1.839-19.412	**0.003**	4.752	1.241-18.191	**0.023**
Gender						
Female	1					
Male	1.716	0.576-5.115	0.333			
BMI						
< 25kg/m^2^	1					
≥ 25kg/m^2^	0.565	0.146-2.187	0.409			
Tumor location						
Head and Neck	1					
Body and Tail	0.608	0.186-1.993	0.608			
Tumor size						
≤2cm	1					
>2cm	4.728	1.002-22.303	**0.050**	0.934	0.137-6.356	0.944
Type						
Nonfunctional PanNENs	1					
Insulinoma	0.090	0.012-0.694	**0.021**	0.360	0.032-4.012	0.406
Pathological grade						
G1 and G2	1					
G3 and NEC	13.393	4.491-39.941	< **0.001**	5.180	1.309-20.493	**0.019**
Clinical stage						
I and II	1					
III and IV	19.688	5.920-65.475	< **0.001**	6.683	1.429-31.263	**0.016**
Hyperlipidemia						
Absence	1					
Presence	0.416	0.128-1.352	0.145			
TC						
<5.2mmol/L	1					
≥5.2mmol/L	0.233	0.030-1.791	0.161			
TG						
<1.70mmol/L	1					
≥1.70mmol/L	0.257	0.033-1.976	0.192			
HDL						
<1mmol/L	1					
≥1mmol/L	0.595	0.163-2.177	0.433			
LDL						
<3.4mmol/L	1					
≥3.4mmol/L	0.038	0.000-24.491	0.322			

HR, hazard ratio; 95%CI, 95% confidence interval; TG, triglyceride; TC, total cholesterol; HDL, high-density lipoprotein; LDL, low-density lipoprotein. The bold indicates the difference was statistically significant (p<0.05).

## 4 Discussion

With the rapid development of the global economy, altered eating habits, and unhealthy lifestyle behaviors, dyslipidemia are one of the most critical challenges of global health problems, over 39% of adults 25 years of age have elevated cholesterol 2008 (24). Numerous chronic illnesses have a significant correlation with HDL. Through encouraging reverse cholesterol transfer, HDL is believed to be essential for protecting against atherosclerosis (25). Additionally, a meta-analysis showed that HDL was associated with a lower chance of having an ischemic stroke (P = 0.004) (26). Lipid metabolism disorder exists in many malignant tumors and influences its occurrence, evolution, and prognosis (8–11). At present, a few previous studies have reported the relationship between dyslipidemia and neuroendocrine neoplasms (NENs). Multicenter research has discovered that obviously lower HDL and TC existed in gastric cancer with neuroendocrine immunophenotypes group in comparison to normal population. Higher TG and HDL may reduce the overall risk of developing gastric cancer with neuroendocrine carcinoma (27). In the rectal neuroendocrine tumors, low HDL level (OR: 1.85, 95% CI: 1.10-3.11) were independent risk factors (28). In this retrospective study, Multiple logistic regression analysis revealed that high levels of HDL are negatively correlated to tumor size, but HDL was not associated with pathological grade or metastasis. Our study also discovers that the serum HDL levels of patients with insulinoma were higher than those of NF-PanNENs.

During the development, growth, and metastasis of tumors, HDL is thought to be protective factor (8, 12, 29, 30). On the other hand, there are less research on HDL and the size of tumors. Our results show a negative association between HDL and tumor size (OR: 0.233, 95% CI: 0.069-0.790, P=0.019). Ana Silva et al. conducted a retrospective analysis of the relationship between colon cancer and Metabolic syndrome. The results demonstrated that non-low HDL patients in 1/2 T-stage were 33.4% higher than low HDL patients (P=0.008) (23). Mariana P. Monteiro et al. discover that IL-6 peritumoral expression was higher in gastro-intestinal NENs of patients with low HDL, and was positively correlated with disease progression (31). Decreased levels of HDL have been associated with increased circulating levels of proinflammatory cytokines such as interleukin 6 and tumor necrosis factor-α receptors, whereas increased levels of HDL are related to raised levels of anti-inflammatory cytokines such as interleukin 10. These proinflammatory cytokines are considered to inhibit apoptosis, which may explain why higher serum HDL concentrations are associated with reduced tumor size. The results of a recent study show that HDL level was an independent associated factor for lymph node metastasis in PanNENs(OR = 0.24, 95%CI: 0.58–0.99) (32).Similar results were seen in a case–control observational study that included 109 gastroenteropancreatic Neuroendocrine tumors patients (33). HDL concentrations in NENs patients who had metastases were 7.45 mg/dl lower than HDL concentrations in NENs patients who did not have metastases(P=0.027). Patients who had elevated serum HDL levels had a reduced chance of developing G2/G3 malignancies (OR: 0.35, 95% CI: 0.12–0.99) (32). As a consequence of their high proliferation activity and potential need for additional cholesterol for membrane formation, advanced PanNENs tend to have reduced amounts of HDL due to increased lipid absorption and lipoprotein loss (34). HDL is a critical regulator of proinflammatory cytokines and oxidative stress. HDL has been shown to play an important role in immune regulation by inhibiting antigen-presenting cells and modulating complement activation, in addition to preventing the production of pro-inflammatory oxidized lipids *via* paraoxonase 1 (35). As one of the key carriers of cholesterol regulation, HDL plays an important role in cancer development through anti-inflammation, antioxidation, immune-modulation, and mediating cholesterol transportation in cancer cells and noncancer cells (29, 36, 37).

In our previous study, we did not find a correlation between changes in blood lipids and prognosis of pancreatic cancer patients (38). Metabolic influence NENs overall outcomes has not been much reported. In digestive NENs, the survival rate of patients with high LDL levels was 4.738 times higher than that of patients with low LDL levels (OR:4.738, 95% CI:1.424-15.772, P= 0.019), but there is not evidently relevance on survival time (39). In well-differentiated gastro-entero-pancreatic NENs with metabolic syndrome,although the median OS of patients with low HDL was higher when compared to those of patients with normal or elevated HDL (207.0 months vs 142.0 months),it didn’t show a statistical difference (40). In this study, we also did not discover any correlation that was statistically significant between dyslipidaemia and mortality. There were multiple reasons that lead to this conclusion. On the one hand, it was possible responsible for the short-term follow-up to restrict access to end events, which may have affected the data analysis. There also exists another possibility that insulinoma secretes a large amount of insulin, which affects glucose metabolism and then lipid metabolism. Therefore, dyslipidemia is the outcome of insulin action but is not a risk factor. Whether there is a correlation between alterations in blood lipid profile and the prognosis of tumors is still debatable. In the future, it is anticipated that high-quality studies with large sample sizes and numerous centers will settle this issue.

Patients with insulinomas are often more obese (43.1% vs 56.9%, P=0.001), probably because of hunger or because preventing hypoglycemia prompts patients eating frequency (41). Blood lipid levels in obese patients are characterized by increased LDL and triglyceride as well as decreased HDL in serum (42, 43). However, in insulinomas, the serum HDL values are significantly higher than in NF-PanNENs (1.306 ± 0.324 vs 1.175 ± 0.315, P=0.006). We speculate that elevated HDL level is due to special glucose metabolism and lipid metabolism in patients with insulinoma. Between glucose and lipid metabolism is much more complex and intimate, the findings of Ibrahim Aslan et al. indicate that insulin analog initiation therapy activates lipid metabolism *via* up-regulating cholesteryl ester transfer protein (CETP) and can increase HDL levels (44, 45).In patients with insulinoma, islet cells can continuously secrete a large amount of insulin, resulting in hypoglycemia. It has been proposed that small amounts of very low-density lipoprotein (VLDL) are synthesized and secreted by the liver in response to decreased levels of carbohydrate-responsive element-binding protein and sterol regulatory element-binding protein due to hypoglycemia, which in turn downregulates CETP expression, thereby facilitating triacylglycerols depletion from cholesteryl esters and its enrichment with HDL (46). Moreover, HDL also increases insulin secretion in pancreatic β cells (19). Through a number of different mechanisms, insulinoma has an impact on the lipid and glucose metabolisms.

A major strength of our study is the first study that the relationship between serum lipid level and clinicopathological characteristics was detailed analyzed, and discussed its effect on prognosis in PanNENs. Our study has several limitations. Firstly, single-center and retrospective studies limit the adaptability of results. Although we developed a rigorous entry form group and utilized scientific classification techniques, potential bias regarding the definition of dyslipidemia and the retrospective design may be the weak points of this study. In addition, the blood lipid level of patients may fluctuate due to many factors, and one test may not accurately reflect the blood lipid level. Although we conduct logistic regression and Cox regression models were executed to revise for confounding factors, we admit the generalizability of the findings could be restricted by a reduced sample number, which would also make it more challenging to find statistically meaningful group differences. Finally, the short follow-up time and curative surgery showing excellent clinical results in PanNENs may affect the accuracy of the results. Multicenter clinical prospective studies with larger sample sizes may draw new conclusions. It is also necessary to carry out further basic research on the correlation between dyslipidemia and patients’ disease progression.In summary, our study showed that the level of HDL increased evidently in patients with insulinoma, and a negative correlation has been observed between tumor size and HDL. Besides, changes in serum lipids as an independent predictor of prognosis cannot be derived from data in PanNENs patients.These results reveal the effect of lipid metabolism on PanNENs. At the same time, our study offers a clinical evidence for the future investigation of lipid metabolism in insulinomas.

## Data availability statement

The original contributions presented in the study are included in the article/**Supplementary Material**. Further inquiries can be directed to the corresponding author.

## Ethics statement

This study was approved by our institutional Ethics Committee and conformed to the provisions of the Declaration of Helsinki.

## Author contributions

H-XZ and Z-YF designed the study, Y-FM performed the statistical analysis and the writing. All authors collected the clinical data and drafted the manuscript. H-XZ and Z-YF critically reviewed the manuscript. All authors read and approved the final manuscript.
