# Supplementary material for: The characteristics of serum lipid spectrum in PanNENs and its correlation with clinicopathological features and prognosis
Source: Front Endocrinol (Lausanne). 2023 Mar 24;14:1137911. doi: 10.3389/fendo.2023.1137911 (PMC10081680; doi:10.3389/fendo.2023.1137911)
Supplement: Supplementary file 1 [file Table_1.docx]

Supplementary Material

**The characteristics of serum lipid spectrum in PanNENs and its correlation with clinicopathological features and prognosis**

Yu-Fan Meng ^† 1^, Zhi-Yao Fan ^† 1^, Jian Yang ^† 1^, Yong-Zheng Li ^1^, Shu-Jie Liu ^1^, Chang-Hao Gao ^1^, Xin Gao^1^, Chao-Yu Pang^1^, Han-Xiang Zhan ^1 *^.

1 Division of Pancreatic Surgery, Department of General Surgery, Qilu hospital, Shandong University, Jinan, Shandong Province, China.

† First authorship

* **Corresponding author:** Han-Xiang Zhan, M.D, PhD. Department of General Surgery, Qilu hospital, Shandong University, Jinan, Shandong. Province, 250012, China. Tel: +86-0531-82166351, Fax: +86-0531-82166009. E-mail: zhanhanxiang@hotmail.com

**Supplementary Table 1** Comparison of clinicopathologic parameters of TG, TC and LDL.

| **Parameters** | **TC<5.2mmol/L**  **(n=171)** | **TC ≥5.2mmol/L**  **(n=14)** | **p-value** | **TG <1.7mmol/L (n=136)** | **TG ≥1.7mmol/L (n=49)** | **p-value** | **LDL <3.4mmol/L (n=159)** | **LDL ≥3.4 mmol/L (n=26)** | **p-value** |
| --- | --- | --- | --- | --- | --- | --- | --- | --- | --- |
| **Type** |  |  | 0.537 |  |  | 0.723 |  |  | 0.688 |
| Nonfunctional PanNENs, n (%) | 78 (78) | 22 (22) |  | 73 (73) | 27 (27) |  | 85(85) | 15(15) |  |
| Insulinoma, n (%) | 63 (74.1) | 22 (25.9) |  | 64 (75.3) | 21(24.2) |  | 74(87.1) | 11(12.9) |  |
| **Age** |  |  | **0.042** |  |  | 0.939 |  |  | 0.643 |
| <60 years, n (%) | 103(73) | 25(56.8) |  | 95(69.3) | 33(68.8) |  | 109(68.6) | 19(73.1) |  |
| ≥60 years, n (%) | 38(27) | 19(43.2) |  | 42(30.7) | 15(31.3) |  | 50(31.4) | 7(26.9) |  |
| **Gender** |  |  | **0.027** |  |  | 0.728 |  |  | 0.226 |
| Female, n (%) | 76(53.9) | 32(72.7) |  | 81(59.1) | 27(56.3) |  | 90(56.6) | 18(69.2) |  |
| Male, n (%) | 65(46.1) | 12(27.3) |  | 56(40.9) | 21(43.8) |  | 69(43.4) | 8(30.8) |  |
| **BMI** |  |  | 0.445 |  |  | 0.245 |  |  | 0.193 |
| < 25kg/m^2^, n (%) | 61(60.4) | 13(52) |  | 58(61.7) | 16(50) |  | 67(60.9) | 7(43.8) |  |
| ≥ 25kg/m^2^, n (%) | 40(39.6) | 12(48) |  | 36(38.3) | 16(50) |  | 43(39.1) | 9(56.3) |  |
| **Tumor location** |  |  | 0.115 |  |  | 0.894 |  |  | 0.823 |
| Head and Neck, n (%) | 54(40.9) | 24(54.5) |  | 58(44.6) | 20(43.5) |  | 68(44.7) | 11(42.3) |  |
| Body and Tail, n (%) | 78(59.1) | 20(45.5) |  | 72(55.4) | 26(56.5) |  | 83(55.3) | 15(57.7) |  |
| **Tumor size** |  |  | 0.533 |  |  | 0.936 |  |  | 0.971 |
| ≤2cm, n (%) | 69(50.4) | 24(55.8) |  | 69(51.5) | 24(52.2) |  | 80(51.6) | 13(52) |  |
| >2cm, n (%) | 68(49.6) | 19(44.2) |  | 65(48.5) | 22(47.8) |  | 75(48.4) | 12(48) |  |
| **Pathological grade** |  |  | 0.283 |  |  | 1 |  |  | 1 |
| G1 and G2, n (%) | 12(91.4) | 36(85.7) |  | 12(90.2) | 43(89.6) |  | 140(89.7) | 23(92) |  |
| G3 and NEC, n (%) | 12(8.6) | 6(14.3) |  | 13(9.8) | 5(10.4) |  | 16(10.3) | 2(8) |  |
| **Distant metastasis** |  |  | 0.571 |  |  | 0.410 |  |  | 1 |
| Absence, n (%) | 12(88.7) | 41(93.2) |  | 12(88.3) | 45(93.8) |  | 142(89.3) | 24(92.3) |  |
| Presence, n (%) | 16(11.3) | 3(6.8) |  | 16(11.7) | 3(6.3) |  | 17(10.7) | 2(7.7) |  |
| **Clinical stage** |  |  | 0.169 |  |  | **0.039** |  |  | 0.531 |
| I and II, n (%) | 11(82.3) | 40(90.9) |  | 111(81) | 45(91.7) |  | 133(83.6) | 23(88.5) |  |
| III and IV, n (%) | 25(17.7) | 4(9.1) |  | 26(19) | 3(6.3) |  | 26(16.4) | 3(11.5) |  |

p-value from Pearson Chi-square Test. The bold indicates the difference was statistically significant (p<0.05).

**Supplementary Table 2** Comparison of clinicopathologic parameters of TG, TC and LDL level.

| **Parameters** | **TC (mmol/L)**  **‾x ± s** | **p-value** | **TG (mmol/L)**  **‾x ± s** | **p-value** | **LDL (mmol/L)**  **‾x ± s** | **p-value** |
| --- | --- | --- | --- | --- | --- | --- |
| **Type** |  | 0.471 |  | 0.576 |  | 0.245 |
| Nonfunctional PanNENs | 4.568±1.186 |  | 1.366±0.826 |  | 2.719±0.813 |  |
| Insulinoma | 4.451±0.980 |  | 1.433±0.789 |  | 2.588±0.690 |  |
| **Age** |  | 0.170 |  | 0.778 |  | 0.588 |
| <60 years | 4.441±1.019 |  | 1.386±0.804 |  | 2.638±0.761 |  |
| ≥60 years | 4.680±1.242 |  | 1.422±0.824 |  | 2.704±0.761 |  |
| **Gender** |  | **0.044** |  | 0.284 |  | 0.482 |
| Female | 4.651±1.057 |  | 1.448±0.926 |  | 2.692±0.784 |  |
| Male | 4.323±1.125 |  | 1.326±0.603 |  | 2.612±0.727 |  |
| **BMI** |  | 0.254 |  | 0.461 |  | 0.254 |
| < 25kg/m^2^ | 4.329±0.923 |  | 1.356±0.892 |  | 2.537±0.720 |  |
| ≥ 25kg/m^2^ | 4.532±1.056 |  | 1.471±0.810 |  | 2.688±0.729 |  |
| **Tumor location** |  | 0.486 |  | 0.979 |  | 0.898 |
| Head and Neck | 4.608±1.221 |  | 1.403±0.798 |  | 2.683±0.777 |  |
| Body and Tail | 4.490±1.015 |  | 1.400±0.831 |  | 2.668±0.763 |  |
| **Tumor size** |  | 0.990 |  | 0.430 |  | 0.449 |
| ≤2cm | 4.528±0.983 |  | 1.446±0.834 |  | 2.622±0.706 |  |
| >2cm | 4.526±1.197 |  | 1.350±0.797 |  | 2.707±0.805 |  |
| **Pathological grade** |  | 0.691 |  | 0.637 |  | 0.874 |
| G1 and G2 | 4.496±1.102 |  | 1.411±0.835 |  | 2.657±0.769 |  |
| G3 and NEC | 4.605±1.117 |  | 1.315±0.614 |  | 2.629±0.690 |  |
| **Distant metastasis** |  | 0.351 |  | **0.002** |  | 0.662 |
| Absence | 4.540±1.093 |  | 1.438±0.831 |  | 2.667±0.744 |  |
| Presence | 4.292±1.116 |  | 1.038±0.431 |  | 2.586±0.900 |  |
| **Clinical stage** |  | 0.096 |  | <**0.001** |  | 0.254 |
| I and II | 4.572±1.089 |  | 1.461±0.850 |  | 2.686±0.740 |  |
| III and IV | 4.203±1.094 |  | 1.051±0.383 |  | 2.511±0.856 |  |

p-value from independent-sample t-test. The bold indicates the difference was statistically significant (p<0.05).

**Supplementary Table 3** Comparison of TG, TC and LDL levels in PanNENs with different clinicopathological parameters

| **Parameters** | **TC**  **(mmol/L)**  **‾x ± s** | **p-value** | **TG**  **(mmol/L)**  **‾x ± s** | **p-value** | **LDL**  **(mmol/L)**  **‾x ± s** | **p-value** |
| --- | --- | --- | --- | --- | --- | --- |
| **Age** |  |  |  |  |  |  |
| **<60 years** |  | 0.560 |  | 0.992 |  | 0.377 |
| Nonfunctional PanNENs | 4.490±1.070 |  | 1.387±0.901 |  | 2.695±0.837 |  |
| Insulinoma | 4.384±0.964 |  | 1.385±0.684 |  | 2.575±0.666 |  |
| **≥60 years** |  | 0.717 |  | 0.336 |  | 0.465 |
| Nonfunctional PanNENs | 4.733±1.406 |  | 1.323±0.649 |  | 2.770±0.769 |  |
| Insulinoma | 4.612±1.017 |  | 1.549±1.003 |  | 2.620±0.758 |  |
| **Gender** |  | 0.686 |  | 0.722 |  | 0.505 |
| **Female** | 4.694±1.091 |  | 1.481±1.000 |  | 2.744±0.819 |  |
| Nonfunctional tional PanNENs | 4.611±1.032 |  | 1.417±0.860 |  | 2.643±0.754 |  |
| Insulinoma |  |  |  |  |  |  |
| **Male** |  | 0.255 |  | 0.117 |  | 0.168 |
| Nonfunctional PanNENs | 4.412±1.233 |  | 1.259±0.570 |  | 2.686±0.781 |  |
| Insulinoma | 4.126±0.790 |  | 1.474±0.635 |  | 2.462±0.543 |  |
| **BMI** |  |  |  |  |  |  |
| **< 25kg/m^2^** |  | 0.645 |  | 0.413 |  | 0.149 |
| Nonfunctional PanNENs | 4.364±0.977 |  | 1.295±0.837 |  | 2.624±0.787 |  |
| Insulinoma | 4.259±0.822 |  | 1.476±0.999 |  | 2.368±0.539 |  |
| **≥ 25kg/m^2^** |  | 0.637 |  | 0.968 |  | 0.459 |
| Nonfunctional PanNENs | 4.624±1.191 |  | 1.477±0.829 |  | 2.787±0.804 |  |
| Insulinoma | 4.479±0.985 |  | 1.467±0.811 |  | 2.630±0.689 |  |
| **Tumor location** |  |  |  |  |  |  |
| **Head and Neck** |  | 0.538 |  | 0.061 |  | 0.678 |
| Nonfunctional PanNENs | 4.669±1.370 |  | 1.500±0.917 |  | 2.709±0.807 |  |
| Insulinoma | 4.487±0.862 |  | 1.210±0.433 |  | 2.630±0.724 |  |
| **Body and Tail** |  | 0.706 |  | 0.078 |  | 0.195 |
| Nonfunctional PanNENs | 4.535±0.977 |  | 1.230±0.728 |  | 2.783±0.850 |  |
| Insulinoma | 4.457±1.050 |  | 1.528±0.886 |  | 2.581±0.685 |  |
| **Tumor size** |  |  |  |  |  |  |
| **≤2cm** |  | 0.522 |  | 0.893 |  | 0.713 |
| Nonfunctional PanNENs | 4.643±0.948 |  | 1.426±0.945 |  | 2.669±0.697 |  |
| Insulinoma | 4.490±0.998 |  | 1.453±0.802 |  | 2.606±0.713 |  |
| >**2cm** |  | 0.326 |  | 0.526 |  | 0.235 |
| Nonfunctional PanNENs | 4.586±1.235 |  | 1.338±0.805 |  | 2.749±0.830 |  |
| Insulinoma | 4.281±0.887 |  | 1.473±0.808 |  | 2.502±0.586 |  |
| **Pathological grade** |  |  |  |  |  |  |
| **G1 and G2** |  | 0.495 |  | 0.523 |  | 0.230 |
| Nonfunctional PanNENs | 4.554±1.201 |  | 1.370±0.866 |  | 2.728±0.840 |  |
| Insulinoma | 4.436±0.992 |  | 1.454±0.806 |  | 2.583±0.686 |  |
| **Distant metastasis** |  |  |  |  |  |  |
| **Absence** |  | 0.388 |  | 0.788 |  | 0.257 |
| Nonfunctional PanNENs | 4.612±1.188 |  | 1.421±0.870 |  | 2.732±0.788 |  |
| Insulinoma | 4.465±0.987 |  | 1.456±0.795 |  | 2.600±0.695 |  |
| **Presence** |  | 0.707 |  | 0.346 |  | 0.496 |
| Nonfunctional PanNENs | 4.335±1.186 |  | 1.080±0.459 |  | 2.649±0.955 |  |
| Insulinoma | 4.060±0.755 |  | 0.817±0.078 |  | 2.250±0.495 |  |
| **Clinical stage** |  |  |  |  |  |  |
| **I and II** |  | 0.199 |  | 0.932 |  | 0.128 |
| Nonfunctional PanNENs | 4.690±1.187 |  | 1.468±0.912 |  | 2.781±0.780 |  |
| Insulinoma | 4.465±0.987 |  | 1.456±0.795 |  | 2.600±0.695 |  |
| **III and IV** |  | 0.816 |  | 0.269 |  | 0.587 |
| Nonfunctional PanNENs | 4.220±1.137 |  | 1.078±0.395 |  | 2.541±0.890 |  |
| Insulinoma | 4.060±0.755 |  | 0.817±0.078 |  | 2.250±0.495 |  |

p-value from independent-sample t-test. The bold indicates the difference was statistically significant (p<0.05).
